# Deep learning–based prediction of compressive strength of eco-friendly geopolymer concrete

**DOI:** 10.1007/s11356-024-33853-2

**Published:** 2024-06-07

**Authors:** Harun Tanyildizi

**Affiliations:** Department of Civil Engineering, Firat University, Elazig, Turkey

**Keywords:** Eco-friendly geopolymer concrete, Compressive strength, Deep LSTM, Machine learning

## Abstract

The greenhouse gases cause global warming on Earth. The cement production industry is one of the largest sectors producing greenhouse gases. The geopolymer is produced with synthesized by the reaction of an alkaline solution and the waste materials such as slag and fly ash. The use of eco-friendly geopolymer concrete decreases energy consumption and greenhouse gases. In this study, the *f*_c_ (compressive strength) of eco-friendly geopolymer concrete was predicted by the deep long short-term memory (LSTM) network model. Moreover, the support vector regression (SVR), least squares boosting ensemble (LSBoost), and multiple linear regression (MLR) models were devised to compare the forecast results of the deep LSTM algorithm. The input variables of the models were used as the mole ratio, the alkaline solution concentration, the curing temperature, the curing days, and the liquid-to-fly ash mass ratio. The output variable of the proposed models was chosen as the compressive strength (*f*_c_). Furthermore, the effects of the input variable on the *f*_c_ of eco-friendly geopolymer concrete were determined by the sensitivity analysis. The *f*_c_ of eco-friendly geopolymer concrete was predicted by the deep LSTM, LSBoost, SVR, and MLR models with 99.23%, 98.08%, 78.57%, and 88.03% accuracy, respectively. The deep LSTM model forecasted the *f*_c_ of eco-friendly geopolymer concrete with higher accuracy than the SVR, LSBoost, and MLR models. The sensitivity analysis obtained that the curing temperature was the most important experimental variable that affected the *f*_c_ of geopolymer concrete.

## Introduction

The most used material in the world is water. The second most used material in the world is concrete. Furthermore, cement production consumes between 12 and 15% of the total industrial energy in the world (Ali et al. 2011). When a ton of cement is produced, 600–800 kg of carbon dioxide arises (Huntzinger and Eatmon 2009; Li et al. 2011; Peng et al. 2013). Today, cement production has a carbon dioxide emission of between 4 and 7% in the world. It is predicted that this emission value in cement production will increase by up to 15% in the next decade (Mahasenan et al. 2003). The geopolymer was first mentioned by Davidovits in 1979 (Davidovits 1979, 1988b, a, 1991). He used kaolinite (Al_2_Si_2_O_5_(OH)) and alkali activators in the main reaction of geopolymerization (Ryu et al. 2013). The carbon dioxide emission of the geopolymer is 60 to 80% less than comparable to Portland cement (Duxson et al. 2007). The geopolymer can be produced using fly ash, slag, metakaolin, red mud, etc. materials. Fly ash (FA) is both a cheap and easily obtainable material (Xu and Deventer 2000; Meesala et al. 2020). So, FA is a good raw material for geopolymer concrete or mortar because fly ash contains high amounts of silicon and aluminum (Zhao et al. 2019). According to conventional concrete, the geopolymer concrete produced using FA has less shrinkage, better performance against sulfate and acid attack, higher chloride ingress resistance, higher freeze–thaw resistance, less alkali-aggregate reaction, and higher resistance under temperature (García-Lodeiro et al. 2007; Tanyildizi and Yonar 2016; Gunasekara et al. 2016; Zhao et al. 2019; Meesala et al. 2020). Also, many factors, including material composition and curing conditions, impact the performance of geopolymer composites (Wang et al. 2024). Ersoy and Çavuş (2023) studied the properties of foam geopolymer composites under different curing conditions. They stated that the high curing temperature increased both the physical and strength qualities of the specimens. Zailani et al. (2024) investigated the effect of the binder/sand ratio on the strength features of geopolymer composites. They found that choosing a binder/sand ratio of 1/2 was optimum in terms of mechanical properties. El-Mir et al. (2023) examined the usability of waste perlite powder in geopolymer composite. They indicated that the 25% replacement of waste perlite powder in the geopolymer composite was optimum. Rohit et al. (2024) researched the strength properties of geopolymer composites containing construction demolition waste. They stated that the use of more than 10% waste material in the samples caused a decrease in the strength properties. Raza et al. (2024) studied the strength properties of geopolymer and cement-based composites. They found that the optimum for building applications was a hybrid cement mortar containing 35% OPC and 5% sodium hydroxide. Thakur and Bawa (2023) investigated the effect of dolomite and ground-granulated blast furnace slag on the mechanical properties of geopolymer composites. They found that using 10% dolomite and ground-granulated blast furnace slag was more cost-effective and showed better strength properties.

Artificial intelligence has been used to forecast the performance of geopolymer or concrete in the last years (Karahan et al. 2008; Nazari and Sanjayan 2015; Lahoti et al. 2017; Tanyildizi 2017, 2018; Soleimani et al. 2018; Akyuncu et al. 2019; Alkroosh and Sarker 2019; Rifaai et al. 2019; Lau et al. 2019; Maleki and Emami 2019; Dao et al. 2019; Ling et al. 2019; Nguyen et al. 2020; Nagajothi and Elavenil 2020; Zhang et al. 2020; Shahmansouri et al. 2021; Kina et al. 2023). Because it is time-consuming and costly to determine the *f*_c_ of eco-friendly geopolymer concrete using an experimental program, the use of artificial intelligence models might speed up the procedure. Kina et al. (2023) prepared the machine learning approaches to guess the *f*_c_ of geopolymer composite. They found that the least-squares boosting model predicted the *f*_c_ of the geopolymer concrete with high accuracy. Eftekhar Afzali et al. (2024) used machine learning algorithms to examine the features of geopolymer concrete. They indicated that machine learning algorithms will help to advance the development of sustainable building materials by facilitating experimental activities, reducing labor and material requirements, and enhancing time efficiency. Latif (2021a) proposed the support vector machine and boosted decision tree regression models to forecast the *f*_c_ of eco-friendly concrete. He found that the support vector machine model had better performance than the boosted decision tree regression model. Tran et al. (2024) tried the *f*_c_ of geopolymer composite by machine learning. They found that the proposed model guessed the *f*_c_ with high accuracy. Lahoti et al. (2017) did a study to forecast the *f*_c_ of metakaolin-based geopolymer concrete. They selected the Naïve Bayes, random forests, and k-nn classifier methods for estimation. They expressed that the k-nn classifier method estimated the *f*_c_ with 83% accuracy. Nazari and Sanjayan (2015) selected the support vector regression (SVR) approach to forecasting the *f*_c_ of geopolymers. They emphasized that the proposed model can forecast the *f*_c_ with 86.91% accuracy. Deep learning modeling has become very popular in recent years (Deng et al. 2018; Jang et al. 2019; Narloch et al. 2019; Abuodeh et al. 2020; Latif 2021b; Tanyildizi 2021, 2024; Chen et al. 2022; Yin et al. 2023). Narloch et al. (2019) tried estimating the *f*_c_ of cement-stabilized rammed Earth with the microstructure images by deep learning. The 4284 images were used in the proposed algorithm. They stated that the deep learning model guessed *f*_c_ with 84% accuracy. Latif (2021b) tried to predict the *f*_c_ of concrete by deep long short-term memory (LSTM) and SVR models. He found that the deep LSTM algorithm predicted the *f*_c_ with higher accuracy than the SVR algorithm. Yin et al. (2023) proposed deep learning models forecasting the *f*_c_ of concrete with waste-rock backfill. They stated that the combined use of the genetic algorithm and deep LSTM forecasted the *f*_c_ of concrete with higher accuracy than the deep LSTM model. Tanyildizi (2024) proposed machine and deep learning hybrid models for guessing the *f*_c_ of engineering cementitious composites. He found that the hybrid deep learning algorithms were better than the machine learning models. Kumar et al. (2024) carried out the comparisons of machine learning and deep learning models in forecasting the *f*_c_ of geopolymer composite. They stated that the performance of the deep learning model was superior to machine learning models. Yao et al. (2024) guessed the carbon emission using deep learning and machine learning algorithms. They stated that the deep learning algorithm made forecasts with higher accuracy.

There is no study estimating the *f*_c_ of eco-friendly geopolymer concrete using the deep LSTM model. So, this present study was made. This study aims to forecast the *f*_c_ of eco-friendly geopolymer concrete using the deep LSTM model. Also, the estimation results of the deep LSTM algorithm were compared to the estimation results of the LSBoost, SVR, and multiple linear regression (MLR) models.

## Data and prediction methodology

### Data

**Fig. 1:**
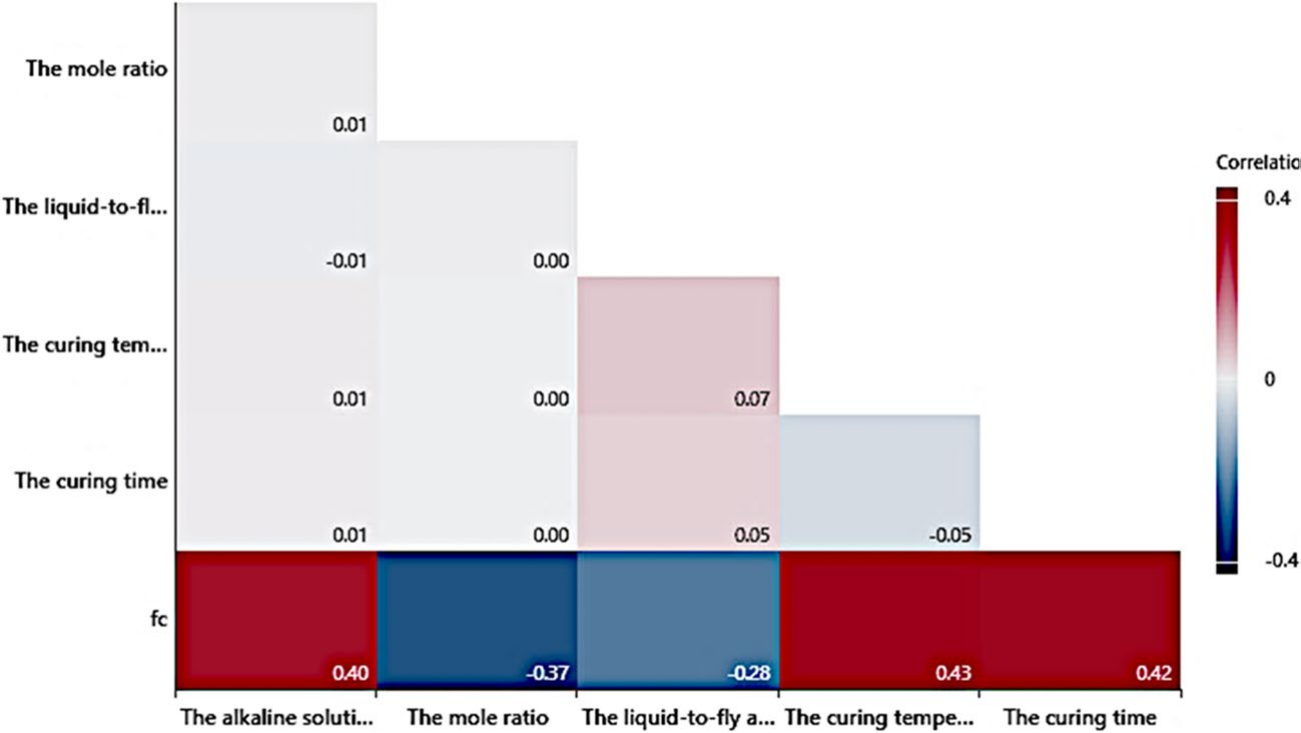
Correlation matrix graph of data

In this study, the *f*_c_ results of eco-friendly geopolymer concrete were taken from the literature (Ling et al. 2019). The database is tabulated in Table 1. It can be seen from Table 1 that the database consisted of the results of 273 specimens for *f*_c_ of eco-friendly geopolymer concrete. The inputs of the models used in forecasting the *f*_c_ of eco-friendly geopolymer concrete were used as the liquid-to-fly ash mass ratio, the mole ratio, the curing temperature, the alkaline solution concentration, and the curing days. The *f*_c_ of the geopolymer concrete is obtained as the output variable. In the literature, descriptive statistical values were given for input and output values (Ibrahim et al. 2023; Golafshani et al. 2024). They are standard deviation, minimum, average, maximum, coefficient of variation, skewness, and kurtosis. The descriptive statistics of inputs and outputs are given in Table 2. Also, Fig. 1. displays a correlation matrix graph of data.

**Table 1:** The *f*_c_ results (Ling et al. 2019)

The experiment number	The alkaline solution concentration (%)	The mole ratio	The liquid-to-fly ash mass ratio	The curing temperature (°C)	The curing time (Days)	*f*_c_ (MPa)
1	15	1	0.33	23	1	3.3
2	15	1	0.33	23	3	6.2
3	15	1	0.33	23	7	9.4
4	15	1	0.33	23	28	28.9
5	15	1	0.33	50	1	22.7
6	15	1	0.33	50	3	29
7	15	1	0.33	50	7	42
8	15	1	0.33	50	28	46.5
9	15	1	0.4	23	1	2.6
10	15	1	0.4	23	3	5
11	15	1	0.4	23	7	8.1
12	15	1	0.4	23	28	28.6
13	15	1	0.4	50	1	19.3
14	15	1	0.4	50	3	27.5
15	15	1	0.4	50	7	36.3
16	15	1	0.4	50	28	46.4
17	20	1	0.33	23	3	9.5
18	20	1	0.33	23	7	19.9
19	20	1	0.33	23	28	57.1
20	20	1	0.33	50	1	42.9
21	20	1	0.33	50	3	53.2
22	20	1	0.33	50	7	65.5
23	20	1	0.33	50	28	75.9
24	20	1	0.4	23	1	1.2
25	20	1	0.4	23	3	7.6
26	20	1	0.4	23	7	17.7
27	20	1	0.4	23	28	50.3
28	20	1	0.4	50	1	29.7
29	20	1	0.4	50	3	39.1
30	20	1	0.4	50	7	50.4
31	25	1	0.33	23	1	4.3
32	25	1	0.33	23	3	26.5
33	25	1	0.33	23	7	44
34	25	1	0.33	23	28	70.1
35	25	1	0.33	50	1	44.7
36	25	1	0.33	50	3	59.5
37	25	1	0.33	50	7	70.6
38	25	1	0.33	50	28	76
39	25	1	0.4	23	1	3.4
40	25	1	0.4	23	3	19.9
41	25	1	0.4	23	7	42.1
42	25	1	0.4	23	28	64.9
43	25	1	0.4	50	1	42.6
44	25	1	0.4	50	3	54.3
45	25	1	0.4	50	7	61.8
46	25	1	0.4	50	28	69
47	15	1	0.5	23	1	1.3
48	15	1	0.5	23	3	3.7
49	15	1	0.5	23	7	6.2
50	15	1	0.5	23	28	24.6
51	15	1	0.5	50	1	13.7
52	15	1	0.5	50	3	22.9
53	15	1	0.5	50	7	26.9
54	15	1	0.5	50	28	39.4
55	15	1	0.6	23	3	3.1
56	15	1	0.6	23	7	5.5
57	15	1	0.6	23	28	22.9
58	15	1	0.6	50	1	10.5
59	15	1	0.6	50	3	16.8
60	15	1	0.6	50	7	22.1
61	15	1	0.6	50	28	38.5
62	20	1	0.33	23	1	3.3
63	20	1	0.4	50	28	61.7
64	20	1	0.5	23	3	4.6
65	20	1	0.5	23	7	14.4
66	20	1	0.5	23	28	40.5
67	20	1	0.5	50	1	19.9
68	20	1	0.5	50	3	24.6
69	20	1	0.5	50	7	40.4
70	20	1	0.5	50	28	49.9
71	20	1	0.6	23	7	12.9
72	20	1	0.6	23	28	30.7
73	20	1	0.6	50	1	9.5
74	20	1	0.6	50	3	13.5
75	20	1	0.6	50	7	25.2
76	20	1	0.6	50	28	32.2
77	25	1	0.5	23	1	4.1
78	25	1	0.5	23	3	14.2
79	25	1	0.5	23	7	38
80	25	1	0.5	23	28	39.8
81	25	1	0.5	50	1	25.5
82	25	1	0.5	50	3	34.3
83	25	1	0.5	50	7	42.1
84	25	1	0.5	50	28	53
85	25	1	0.6	23	3	9.7
86	25	1	0.6	23	7	28.1
87	25	1	0.6	23	28	32.1
88	25	1	0.6	50	1	15.2
89	25	1	0.6	50	3	20
90	25	1	0.6	50	7	27.7
91	25	1	0.6	50	28	39.5
92	15	1	0.5	23	1	1.1
93	15	1.5	0.33	23	1	2.7
94	15	1.5	0.33	23	3	4.7
95	15	1.5	0.33	23	7	7
96	15	1.5	0.33	23	28	11.1
97	15	1.5	0.33	50	1	11.7
98	15	1.5	0.33	50	3	16.6
99	15	1.5	0.33	50	7	22
100	15	1.5	0.33	50	28	28
101	15	1.5	0.4	23	1	1.5
102	15	1.5	0.4	23	3	3.1
103	15	1.5	0.4	23	7	4.5
104	15	1.5	0.4	23	28	8.3
105	15	1.5	0.4	50	1	8.2
106	15	1.5	0.4	50	3	11.9
107	15	1.5	0.4	50	7	14.1
108	15	1.5	0.4	50	28	18.6
109	20	1.5	0.33	23	3	10
110	20	1.5	0.33	23	7	14.3
111	20	1.5	0.33	23	28	22.8
112	20	1.5	0.33	50	1	20.3
113	20	1.5	0.33	50	3	27
114	20	1.5	0.33	50	7	30.5
115	20	1.5	0.33	50	28	32.8
116	20	1.5	0.4	23	1	2.6
117	20	1.5	0.4	23	3	6.5
118	20	1.5	0.4	23	7	10.3
119	20	1.5	0.4	23	28	20.3
120	20	1.5	0.4	50	1	15.9
121	20	1.5	0.4	50	3	22.4
122	20	1.5	0.4	50	7	25.6
123	20	1.5	0.4	50	28	29.4
124	25	1.5	0.33	23	3	17.3
125	25	1.5	0.33	23	7	24.4
126	25	1.5	0.33	23	28	40.8
127	25	1.5	0.33	50	1	36.1
128	25	1.5	0.33	50	3	45.1
129	25	1.5	0.33	50	7	52.7
130	25	1.5	0.33	50	28	59.7
131	25	1.5	0.4	23	1	2.5
132	25	1.5	0.4	23	3	9.6
133	25	1.5	0.4	23	7	16.6
134	25	1.5	0.4	23	28	32.6
135	25	1.5	0.4	50	1	25.4
136	25	1.5	0.4	50	3	35.7
137	25	1.5	0.4	50	7	44.3
138	15	1.5	0.5	23	3	2.2
139	15	1.5	0.5	23	7	3.5
140	15	1.5	0.5	23	28	6.2
141	15	1.5	0.5	50	1	5.2
142	15	1.5	0.5	50	3	8.4
143	15	1.5	0.5	50	7	10.8
144	15	1.5	0.5	50	28	13.8
145	15	1.5	0.6	23	3	1.3
146	15	1.5	0.6	23	7	2
147	15	1.5	0.6	23	28	3.5
148	15	1.5	0.6	50	1	3.3
149	15	1.5	0.6	50	3	5.6
150	15	1.5	0.6	50	7	8
151	15	1.5	0.6	50	28	12
152	20	1.5	0.33	23	1	4.3
153	20	1.5	0.5	23	3	3.7
154	20	1.5	0.5	23	7	6
155	20	1.5	0.5	23	28	12.8
156	20	1.5	0.5	50	1	8.5
157	20	1.5	0.5	50	3	16
158	20	1.5	0.5	50	7	19.7
159	20	1.5	0.5	50	28	25.8
160	20	1.5	0.6	23	3	2.2
161	20	1.5	0.6	23	7	3.9
162	20	1.5	0.6	23	28	10
163	20	1.5	0.6	50	1	7.4
164	20	1.5	0.6	50	3	12.1
165	20	1.5	0.6	50	7	16.4
166	20	1.5	0.6	50	28	24
167	25	1.5	0.33	23	1	8
168	25	1.5	0.4	50	28	54.2
169	25	1.5	0.5	23	3	4.6
170	25	1.5	0.5	23	7	13.8
171	25	1.5	0.5	23	28	28.6
172	25	1.5	0.5	50	1	22
173	25	1.5	0.5	50	3	31.5
174	25	1.5	0.5	50	7	38.9
175	25	1.5	0.5	50	28	46.8
176	25	1.5	0.6	23	7	6.3
177	25	1.5	0.6	23	28	24.3
178	25	1.5	0.6	50	1	9.2
179	25	1.5	0.6	50	3	19.1
180	25	1.5	0.6	50	7	26.3
181	25	1.5	0.6	50	28	36.9
182	15	2	0.33	23	1	2.6
183	15	2	0.33	23	3	4.7
184	15	2	0.33	23	7	6.9
185	15	2	0.5	23	1	1.2
186	15	2	0.5	23	3	2.5
187	15	2	0.5	23	7	3.8
188	15	2	0.33	23	28	14.6
189	15	2	0.33	50	1	10.6
190	15	2	0.33	50	3	15.8
191	15	2	0.33	50	7	15.8
192	15	2	0.33	50	28	25.5
193	15	2	0.4	23	1	1.4
194	15	2	0.4	23	3	2.8
195	15	2	0.4	23	7	4.2
196	15	2	0.4	23	28	6.6
197	15	2	0.4	50	1	5.1
198	15	2	0.4	50	3	8.9
199	15	2	0.4	50	7	12.8
200	15	2	0.4	50	28	18.6
201	20	2	0.33	23	3	7.3
202	20	2	0.33	23	7	11.8
203	20	2	0.33	23	28	21.9
204	20	2	0.33	50	1	14.6
205	20	2	0.33	50	3	20.2
206	20	2	0.33	50	7	25.7
207	20	2	0.33	50	28	48.2
208	20	2	0.4	23	1	2.4
209	20	2	0.4	23	3	4.6
210	20	2	0.4	23	7	6.5
211	20	2	0.4	23	28	14.2
212	20	2	0.4	50	1	12.3
213	20	2	0.4	50	3	19.6
214	20	2	0.4	50	7	24.6
215	20	2	0.4	50	28	36
216	25	2	0.33	23	3	11.9
217	25	2	0.33	23	7	19.3
218	25	2	0.33	23	28	26.9
219	25	2	0.33	50	1	23.2
220	25	2	0.33	50	3	35.8
221	25	2	0.33	50	7	40.5
222	25	2	0.33	50	28	49.7
223	25	2	0.4	23	1	2.7
224	25	2	0.4	23	3	8
225	25	2	0.4	23	7	13.1
226	25	2	0.4	23	28	25.4
227	25	2	0.4	50	1	19
228	25	2	0.4	50	3	30.6
229	25	2	0.4	50	7	39.4
230	25	2	0.4	50	28	45.2
231	15	2	0.5	23	28	4.8
232	15	2	0.5	50	1	6.1
233	15	2	0.5	50	3	9.1
234	15	2	0.5	50	7	11.5
235	15	2	0.5	50	28	14.9
236	15	2	0.6	23	3	1.4
237	15	2	0.6	23	7	2
238	15	2	0.6	23	28	3.4
239	15	2	0.6	50	1	4.1
240	15	2	0.6	50	3	5.9
241	15	2	0.6	50	7	8
242	15	2	0.6	50	28	13.1
243	20	2	0.33	23	1	3.6
244	20	2	0.5	23	3	2.8
245	20	2	0.5	23	7	4.7
246	20	2	0.5	23	28	10.3
247	20	2	0.5	50	1	8.6
248	20	2	0.5	50	3	13.6
249	20	2	0.5	50	7	17.6
250	20	2	0.5	50	28	31.9
251	20	2	0.6	23	3	1.1
252	20	2	0.6	23	7	2.6
253	20	2	0.6	23	28	6.7
254	20	2	0.6	50	1	3.3
255	20	2	0.6	50	3	9.2
256	20	2	0.6	50	7	12.3
257	20	2	0.6	50	28	18.5
258	25	2	0.33	23	1	4.6
259	25	2	0.5	23	1	1.2
260	25	2	0.5	23	3	4.5
261	25	2	0.5	23	7	7.6
262	25	2	0.5	23	28	19.4
263	25	2	0.5	50	1	11.8
264	25	2	0.5	50	3	21.7
265	25	2	0.5	50	7	31.8
266	25	2	0.5	50	28	32.1
267	25	2	0.6	23	3	1.8
268	25	2	0.6	23	7	4.2
269	25	2	0.6	23	28	15.3
270	25	2	0.6	50	1	7.2
271	25	2	0.6	50	3	19.4
272	25	2	0.6	50	7	23.1
273	25	2	0.6	50	28	25.5

**Table 2:** The statistical descriptive values of inputs and outputs

	Standard deviation	Minimum	Average	Maximum	Coefficient of variation	Skewness	Kurtosis
The curing days (day)	10.88	1	10.22	28	20.6	0.95	− 0.93
The alkaline solution concentration (%)	4.11	15	19.96	25	27.42	0.01	− 1.52
The curing temperature (°C)	13.50	23	37.24	50	22.32	− 0.11	− 2
The liquid-to-fly ash mass ratio	0.10	0.33	0.45	0.6	36.26	0.26	− 1.34
*f*_c_ (MPa)	16.95	1.1	20.24	76	106.46	1.1	0.67
The mole ratio	0.41	1	1.5	2	83.76	0	− 1.52

### The methodology of long short-term memory network

**Fig. 2:**
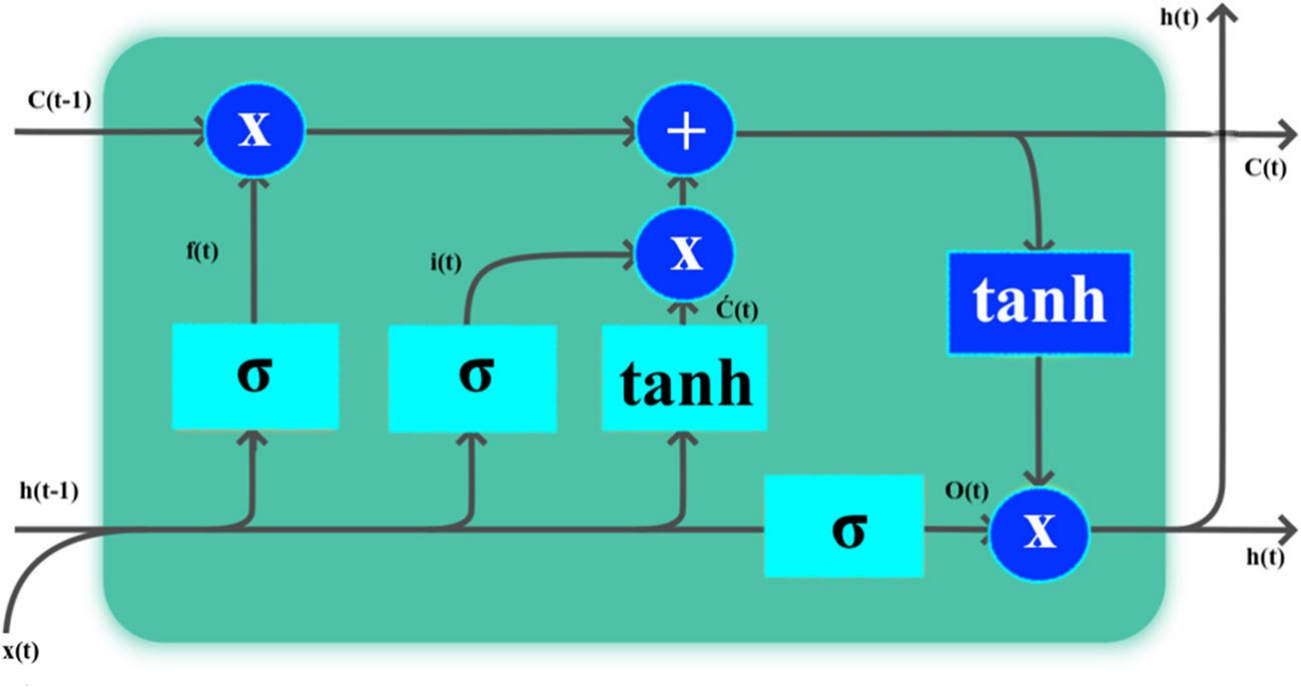
The illustration of the LSTM structure

The deep LSTM was recommended by Hochreiter and Schmidhuber (Hochreiter and Schmidhuber 1997). The LSTM that occurs input (a sequence input layer feeds data into the neural network in the form of a sequence or time series), forget (the forget determines which information is important and which may be disregarded), and output (the value of the next hidden state is calculated by the output; this state includes data from earlier inputs) is a Recurrent Neural Networks. The illustration of LSTM structure is illustrated in Fig. 2.

Figure 2 displays the data flow at time step *t*. Also, this graphic displays how the gates forget, update, and output cell and hidden states. The LSTM occurred in three steps. Firstly, it is calculated which information to delete by x(*t*) and h(*t*-1). This is determined by Eq. 1.

1 $${f}_{t}= \sigma ({W}_{(f,x)}x\left(t\right)+{W}_{(f,h)}{h}_{(t-1)}+ {b}_{f}$$

*t*: timestep
*f*_*t*_: forget get at *t*
*W*: the weights
*x*(*t*): input at time *t*
*h*(*t*-1): the output of the previous state
*b*_*f*_: the biases

Secondly, the input layer for the new information is activated. The information is restructured using the sigmoid function in Eq. 2. Following, the candidate information, which occurs with the new information, is obtained by the tanh function in Eq. 3.

2 $$i\left(t\right)=\sigma ({W}_{(f,x)}x\left(t\right)+{W}_{(f,h)}{h}_{(t-1)}+ {b}_{i}$$

3 $$\boldsymbol{\acute{C} }\left(t\right)=tanh({W}_{(c,x)}x\left(t\right)+{W}_{(c,h)}{h}_{(t-1)}+ {b}_{c}$$

Then, the new information is formed using Eq. 4.

4 $${C}_{t}=C\left(t-1\right)f\left(t\right)+i(t)\boldsymbol{\acute{C} }\left(t\right)$$

Lastly, the output is determined by Eqs. 5 and 6.

5 $$O(t)=\sigma ({W}_{(o,x)}x\left(t\right)+{W}_{(o,h)}{h}_{(t-1)}+ {b}_{o}$$

6 $$h\left(t\right)=O(t)\text{tanh}(C(t))$$

where

*C*_*t*_: value produced by tanh,
*i*_*t*_: input gate at *t*,
*b*: bias vector,
*W*_*c*_: weight matrix of tanh operator between cell state information and network output,
*W*_*o*_: weight matrix of output gate,
*O*(*t*): output gate at *t*, and
*h*(*t*): LSTM output.

Then, bias parameters (*b*) and weight parameters (W) are learned by the model in a way that minimizes the difference between the actual training values and the LSTM output values (Hochreiter and Schmidhuber 1997; Gers et al. 2003; Liu et al. 2019).

### The methodology of support vector regression

The SVR was recommended by Vapnik (2000). The non-linear SVR tries to find a regression function in hyperspace stated by *f* (*x*) = *w* ^T^ϕ (*x*) + *b*. The eq. is calculated by the “ϵ-insensitive” loss function. The below equations are expressed non-linear SVR.

7 $${}_{w,b, \xi ,{\xi }^{*}}{}^{min}=\left({~}^{1}\!\left/ \!{~}_{2}\right.\right){\parallel w\parallel }^{2}+C\left({e}^{T}\xi +{e}^{T}{\xi }^{*}\right)$$

8 $$s.t. (\varnothing \left(A\right)w+eb)-Y\le e\in +\xi ,\xi \ge 0e$$

9 $$Y-\left(\varnothing \left(A\right)w+eb\right)\le e\in +{\xi }^{*},{\xi }^{*}\ge 0e$$

where

*C*: a predetermined parameter. Also, it is an adjustment factor that offers the balance between the adaptation of errors and the flatness of the regression function,
*E*: the unit vector,
ξ and ξ*: slack variables that specify whether specimens entered the ϵ-tube or not.

When using the Lagrange multipliers α and α*, the below Eqs. 10–12 are used.

10 $$\begin{array}{c}max\\ \alpha ,{\alpha }^{*}\end{array}-\left({~}^{1}\!\left/ \!{~}_{2}\right.\right)({\alpha }^{*}-\alpha {)}^{T}K\left(A,{A}^{T}\right)\left({\alpha }^{*}-\alpha \right)+{Y}^{T}\left({\alpha }^{*}-\alpha \right)+\in {e}^{T}({\alpha }^{*}+\alpha )$$

11 $$s.t. {e}^{T}\left({\alpha }^{*}+\alpha \right)=\text{0,0}e\le \alpha ,{\alpha }^{*}\le Ce$$

lastly

12 $$f\left(x\right)=\sum_{i=1}^{n}\left({\alpha }^{*}-\alpha \right)\text{K}\left({x}_{i},\text{x}\right)+b$$

where

α and α* = Lagrange multipliers,

*A* = the input of the training,

*Y* = the output of the training,

*b* = bias parameters,

*K*(*x*_*i*_, *x*) = Kernel function, and

*f*(*x*) = the SVR equation (Burges 1998; İNCE et al. 2016).

### Multiple linear regression

The method of expressing the linear relationship between a dependent variable (*y*) and an independent single variable *x* by a mathematical formula is known as simple linear regression. MLR is a method used to express the relationship between more than one independent variable (*x*_*n*_) and the dependent variable (*y*) (Cohen 2013). The mathematical model showing the relationship in multivariate regression analysis was expressed with the following equation for *m* independent variables.

13 $$Y={h}_{o}+{h}_{1}{x}_{1}+{h}_{2}{x}_{2}+...\dots \dots \dots \dots ..+{h}_{m}{x}_{m}+t$$

where

*Y*: The dependent variable,
*X*: The independent variable,
*h*_0_: The y-axis intercept of the regression curve,
*m*: The number of input parameters,
*h*_*m*_*xm*: The regression coefficient of the independent variable, and
*t*: The error term.

### Least squares boosting ensemble

The gradient-boosting ensemble method includes a restricted collection of frail learners and a meta-learner that gives weights to each learner and then aggregates their estimator findings by voting techniques to obtain a higher forecast result. Least-squares boosting (LSBoost) is a method that uses least squares as its loss criterion (Alajmi and Almeshal 2021). The LSBoost method was first used by Friedman (2001). Friedman suggested using the following equations for the LSBoost method. Friedman (2001) stated that explainable variables (*x*_*i*_ and *y*_*i*_) and the number of iterations (*H*) should be defined. Then, the training set, a loss function, and a regression function are defined using the following equations, respectively.

14 $${\left\{({x}_{i}\left.,{y}_{i}\right\}\right.}_{i=1}^{n}$$

15 $$L\left(y,F\right)=\frac{{(y-F)}^{2}}{2}$$

16 $${F}_{m}(x)$$

Initialize ${F}_{0}\left(x\right)=\overline{y }$; lastly, it is estimated using the following equations.

17 $$\text{For} t=1 \text{to} H \text{do}: {\widetilde{y}}_{i}-{F}_{m-1}\left({x}_{i}\right) \text{for} I=\text{1,2},\dots ,N$$

18 $$\left({\rho }_{t},{\alpha }_{t}\right)={argmin}_{\rho ,\alpha }\sum_{i=1}^{N}{[{\widetilde{y}}_{i}-\rho h({x}_{i};\alpha )]}^{2}$$

19 $${F}_{t}\left(x\right)={F}_{t-1}\left(x\right)+{\rho }_{t}h(x;{\alpha }_{t})$$

where

*x*_*i*_ and *y*_*i*_ = explainable variables,

*H* = the number of iterations,

${\left\{({x}_{i}\left.,{y}_{i}\right\}\right.}_{i=1}^{n}$= the training set,

*L*(*y*, *F*) = a loss function,

*F*_*m*_*(x)* = the regression function,

*h* = activation function,

*α* = a sequence of pseudo-random U ([0; 1]) numbers, and

*ρ* = learning rate (0< $$\rho$$ <1).

### Sensitivity analysis

Sensitivity analysis is a good method to find the effect of experimental variables on the experimental result (Yang and Zhang 1997). The cosine amplitude method in the sensitivity analysis of this study was used. Ross (2010) stated that the cosine amplitude method is a good method to define the effect of experimental parameters on the result. It has a collection of data specimens using n data specimens.

20 $$X=[{x}_{1},{x}_{2},{x}_{3},{x}_{4}\dots \dots \dots \dots \dots \dots ..{x}_{n}]$$

Each of the elements, *x*_*i*_, in the data array *X* is itself a vector of length *m*.

21 $${X}_{i}=[{x}_{i1},{x}_{i2},{x}_{i3},{x}_{i4}\dots \dots \dots \dots \dots \dots ..{x}_{im}]$$

*x*_*j*_ and *x*_*i*_ are the dependent and the independent variables in the data. Thus, the following equation is used to determine the similarity of each element (Khandelwal and Singh 2007)

22 $${r}_{ij}=\frac{{\sum }_{k=1}^{m}{x}_{ik}{x}_{jk}}{\sqrt{{\sum }_{k=1}^{m}{x}_{ik}^{2}{\sum }_{k=1}^{m}{x}_{jk}^{2}}}$$

### Assessments of forecast models

Performance metrics are used to establish the performance of machine learning and deep learning models in the literature (Botchkarev 2018; Solhmirzaei et al. 2020; Steurer et al. 2021; Imik Tanyildizi and Tanyildizi 2022; Plevris et al. 2022; Kina et al. 2022; Naser and Alavi 2023; Turk et al. 2023). In this study, performance metrics were utilized to assess the prediction abilities of deep learning and machine learning models. These metrics are mean square error (MSE), root-mean-square-error (RMSE), the peak signal-to-noise ratio (PSNR), mean absolute percentage error (MAPE), and normalized root-mean-square error (NRMSE). The mathematical expressions of these metrics are given in Eqs. 23–28.

23 $$MSE=\frac{\sum_{i=1}^{n}{\left({y}_{f}- {y}_{a}\right)}^{2}}{n}$$

24 $${R}^{2}=1- \frac{\sum_{i=1}^{n}{\left({y}_{f}- {y}_{a}\right)}^{2}}{\sum_{i=1}^{n}{\left({y}_{a}- {y}_{f}\right)}^{2}}$$

25 $$PSNR=10 {log}_{10}\frac{{R}^{2}}{MSE}$$

26 $$RMSE=\sqrt{\frac{\sum_{i=1}^{n}{\left({y}_{f}- {y}_{a}\right)}^{2}}{n}}$$

27 $$MAPE=\frac{1}{n}\left(\sum_{i=1}^{n} \left|\frac{{y}_{f}- {y}_{a}}{{y}_{f}}\right| \right)* 100$$

28 $$NRMSE=\frac{RMSE}{\sigma }$$

In Eqs. 23–28, $n$ is the whole number of datasets. Also, ${y}_{f}$ and ${y}_{r}$ are the forecasted and actual results, respectively.

## Results

### The results of the deep LSTM model

**Fig. 3:**
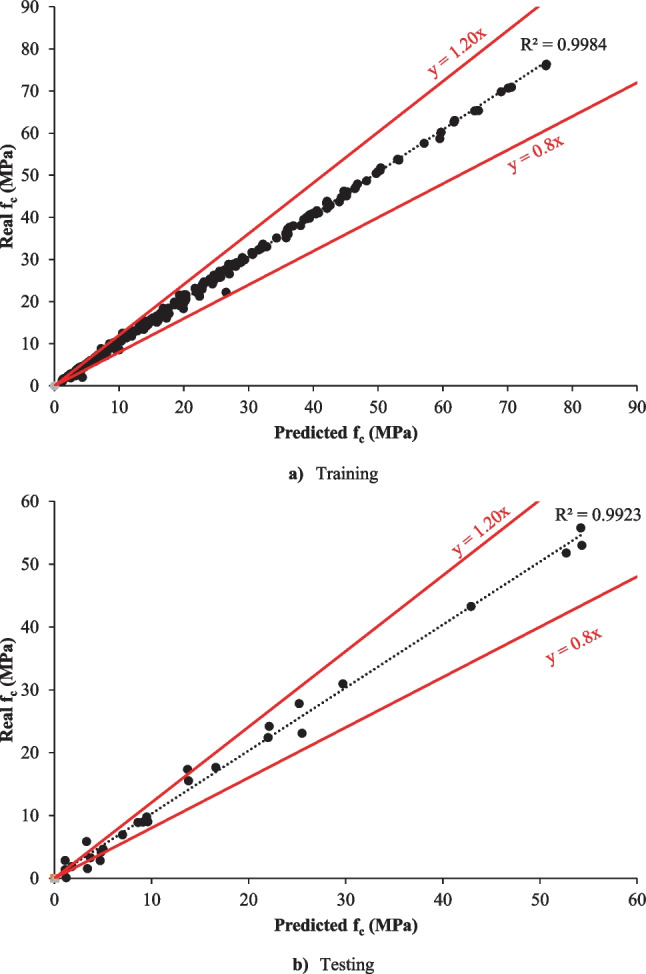
The training and testing results of the deep LSTM model. **a** Training. **b** Testing

**Fig. 4:**
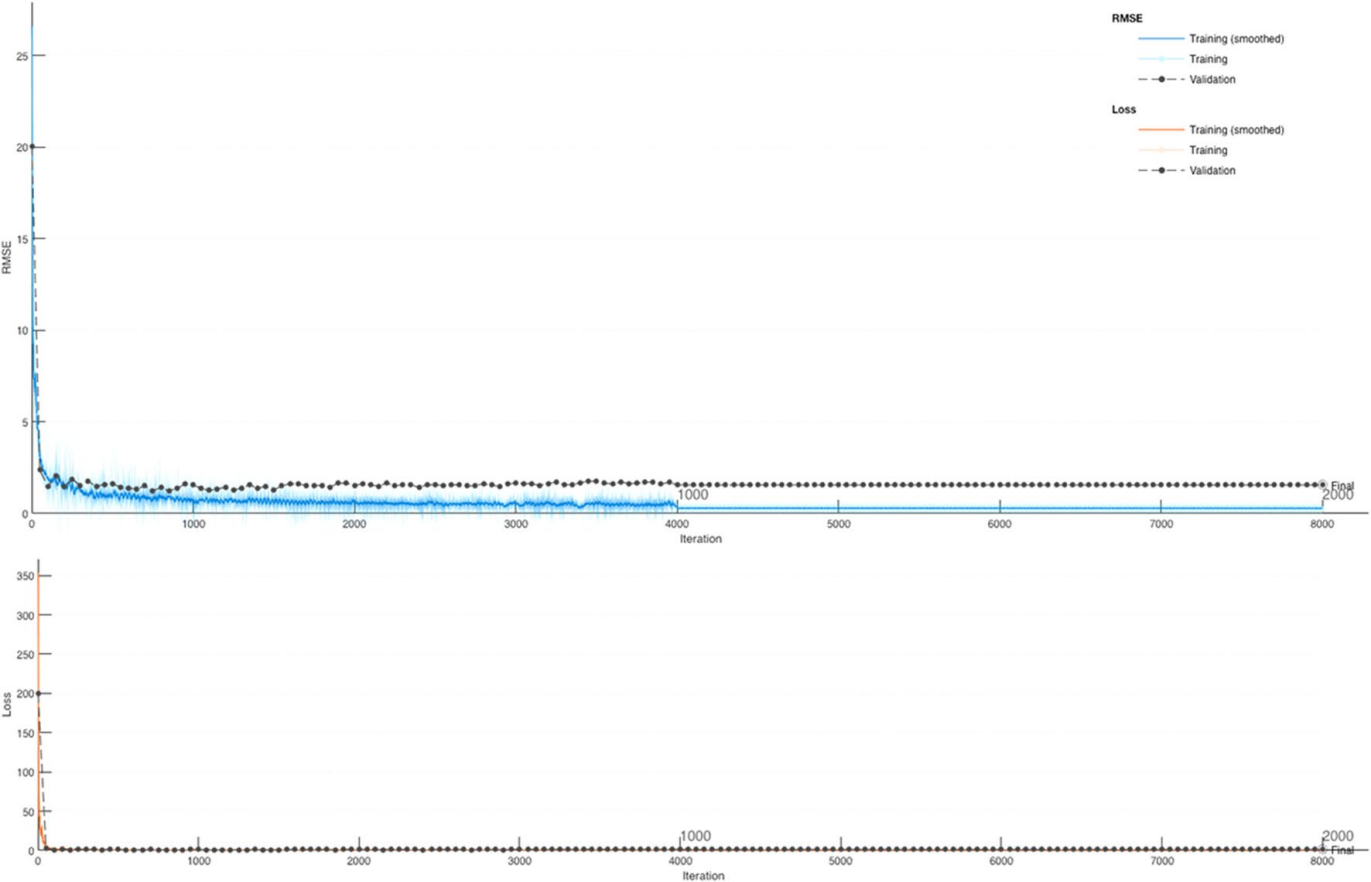
The training process of the deep LSTM model

In this present section, the deep LSTM model was developed to forecast the *f*_c_ of eco-friendly geopolymer concrete. In this study, one output and five inputs for *f*_c_ were selected in the deep LSTM model. Ninety percent of all data was used for training in the model. Ten percent of all data was selected for testing. The fully connected layer, the output size of the bidirectional LSTM layer, the gradient threshold, and the mini-batch size were 1, 200, 1.2, and 30, respectively. The training technique used the “adam.” The learning rate and the initial learning rate were selected with a dropped factor of 0.000001 during the training with 100 epoch periods and 0.1. The selected parameters of the proposed algorithm were obtained by an empirical approach. The deep LSTM forecast results and the training progress for the *f*_c_ of geopolymer concrete are given in Figs. 3 and 4.

It was shown in Fig. 3 that the deep LSTM algorithm guessed the *f*_c_ of eco-friendly geopolymer with 99.84% and 99.23% accuracy for training and testing. Ling et al. (2019) designed an artificial neural network (ANN) algorithm estimating the same data. They found that the proposed ANN algorithm guessed the *f*_c_ of eco-friendly geopolymer concrete with 96.1% accuracy. Moreover, MSE, RMSE, PSNR, MAPE, and NRMSE were selected to identify the performance of the deep LSTM model. MSE and RMSE are also the most commonly used statistical tools to find the difference between the real and forecasted values. The lower these values are, the performance of the model is considered better than other models. NRMSE analyzes the difference between the forecasted values by a mode and the real values. The lower this value, the performance of the model is considered better than other models. MAPE is the relative average vertical distance between target and output values in percentage. The lower this distance, the performance of the model the better than other models. PSNR is the ratio between the maximum possible power of a signal and the power of noise that influences the quality. The larger this ratio, the better the performance of the model than the other models. *R* squared (*R*2) symbolizes the proportion of the variance for the dependent variable *y* that is explained by the independent variables. If the *R*2 of a model is 1, then 100% of the observed variation can be explained by the model’s specifications. Therefore, it is desired to be close to 1. It must be the higher PSNR, lower MSE, NRMSE, lower RMSE, and lower MAPE that the model has good performance. The MSE, the PSNR, the RMSE, the NRMSE, and MAPE values for the training of *f*_c_ of eco-friendly geopolymer concrete were obtained 0.7042, 46.65, 0.8392, 0.0112, and 5.2378, respectively.

The PSNR, MSE, NRMSE, RMSE, and MAPE values for the testing phase were 44.5864, 2.2617, 0.0283, 1.5039, and 224874, respectively. There are very few articles predicting the *f*_c_ of concrete by deep learning methods. Jang et al. (2019) developed a deep learning algorithm forecasting the *f*_c_ of concrete. They used microscope images of concrete in the estimation model. They mentioned that these images could be used to predict *f*_c_. Abuodeh et al. (2020) chose the deep learning technique to guess the *f*_c_ of concrete with high strength. They used the results of 110 samples in the model. They found that the *f*_c_ can be guessed with 80.1% accuracy. Chen et al. (2022) used the deep LSTM and SVR models to guess the mechanical features of concrete. They emphasized that the deep LSTM algorithm forecasted the strength features of concrete with higher accuracy (99.7%) than the SVR algorithm. In this study, the deep LSTM algorithm was proposed to forecast the *f*_c_ of eco-friendly geopolymer concrete with high accuracy.

### The results of the support vector regression model

**Fig. 5:**
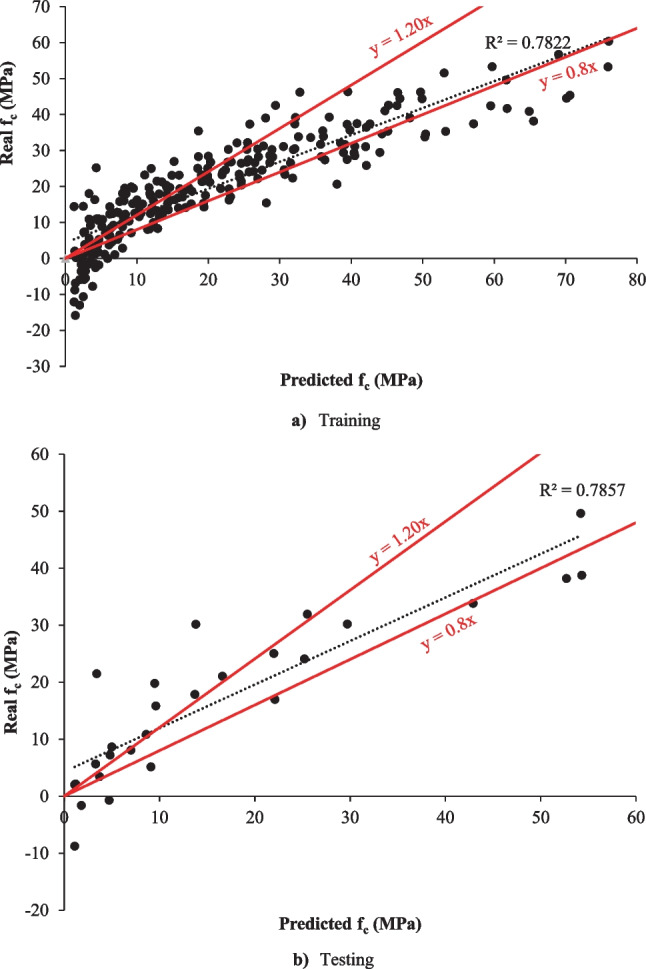
The training and testing results of the SVR model. **a** Training. **b** Testing

**Fig. 6:**
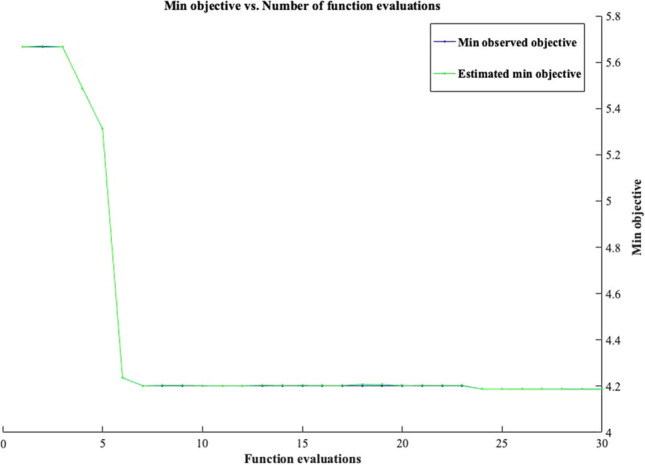
The minimum objective number of function evaluations of the SVR model

In this study, the *f*_c_ of eco-friendly geopolymer concrete was predicted by the SVR algorithm. Similar to the deep LSTM algorithm, the same inputs and outputs were selected in the SVR algorithm. The 10% and 90% of all data were used for testing and training in the model. This study used hyperparameter optimization in the SVR model. As a result of hyperparameter optimization used in SVR, the box constraint, kernel scale, and epsilon values were found to be 0.0042986, 0.0025956, and 2.1922. The Box constraint optimizable hyperparameter combines the preset SVM models’ Box constraint mode and Manual box constraint advanced options. The Kernel scale optimizable hyperparameter includes the preset SVM models’ Kernel scale mode and Manual kernel scale advanced options (MATLAB 2019). The SVR results and the minimum objective number of function evaluations for the *f*_c_ of eco-friendly geopolymer concrete are given in Figs. 5 and 6.

As can be shown in Fig. 5, the SVR model forecasted the *f*_c_ of eco-friendly geopolymer concrete with 78.22% and 78.57% accuracy for training and testing, respectively. Similar to the deep LSTM, the MSE, MAPE, NRMSE, RMSE, and PSNR were used to evaluate the SVR model. The MAPE, RMSE, PSNR, NRMSE, and MSE values for the training of *f*_c_ of eco-friendly geopolymer concrete were obtained as 76.6235, 7.9593, 30.1133, 0.1064, and 63.3505, respectively. The MAPE, RMSE, PSNR, NRMSE, and MSE values for the testing phase were 99.6202, 7.6908, 30.4114, 0.1446, and 59.1481, respectively. When these results of the SVR algorithm are compared to the deep LSTM algorithm, the deep LSTM can be found to be superior. Ling et al. (2019) forecasted the same *f*_c_ of eco-friendly geopolymer concrete with 96.1% accuracy. The proposed SVR model guessed the *f*_c_ with lower accuracy than the ANN model. Rahmati and Toufigh (2022) used the SVR and ANN models to guess the *f*_c_ of geopolymer concrete subjected to high temperatures. They stated that the SVR model predicted better the experimental results than the ANN model. Maleki and Emami (2019) developed an SVR model estimating the *f*_c_ of fly ash-based geopolymer concrete. They stated that the model forecasted the *f*_c_ with 84% accuracy. Wu et al. (2023) evaluated the performance of the SVR algorithm and experimental results of geopolymer mortar exposed to seawater. They mentioned that SVR had a higher performance than ANN in predicting experimental results of geopolymer mortar exposed to seawater.

### The results of the multiple linear regression model

In the present section, multiple linear regression (MLR) was used to estimate the *f*_c_ of eco-friendly geopolymer concrete. The equations obtained from the MLR were given below.

12 $${f}_{c}=1.7208-2.674\times M-0.565\times A-11.43\times L-1.1692\times T-0.4033\times C$$

where

*f*_c_: The compressive strength,
*M*: The mole ratio,
*T*: The curing temperature,
*L*: The liquid-to-fly ash mass ratio,
*A*: The alkaline solution concentration, and
*C*: The curing days.

**Fig. 7:**
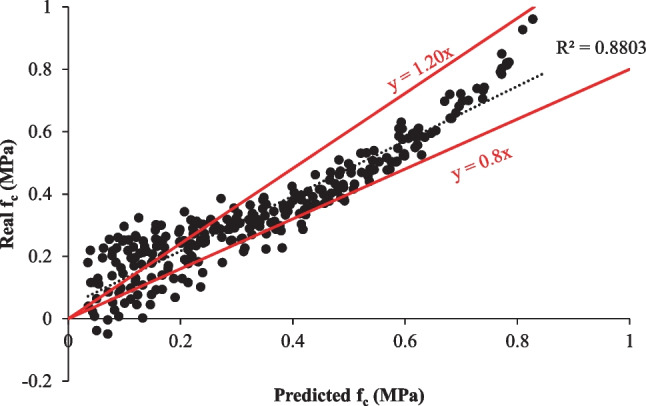
The results of the MLR model

The MLR model results are given in Fig. 7. Figure 7 shows that the MLR model forecasted the *f*_c_ of eco-friendly geopolymer concrete with 88.03% accuracy. The MLR model forecasted the results with less accuracy than the SVR and deep LSTM models. Amin and Nasier (2018) statistically examined the properties of fly ash-based geopolymer concrete. They devised the stepwise multiple linear regression model for *f*_c_. They said that the water-to-powder ratio affected the *f*_c_ by 90%. Kazemian et al. (2015) used an MLR model to forecast the *f*_c_ of geopolymer mortar. They stated that the model guessed the *f*_c_ with 82.36% accuracy. Demir and Derun (2019) investigated the properties of gold mine waste-based geopolymer. Furthermore, they devised an MLR model for the reaction degree. They mentioned that the accuracy of their model was 80.94%. Lee et al. (Lee and Lee 2020) suggested a model predicting the setting time and *f*_c_ of concrete using ultrasonic pulse velocity. They said that the proposed MLR model could be found in the experimental results with a 10% error. In the literature, the accuracy of MLR models has been found to be between 90 and 80%. In this study, it was found to be 88.03%, similar to the results in the literature.

### The results of the LSBoost model

**Fig. 8:**
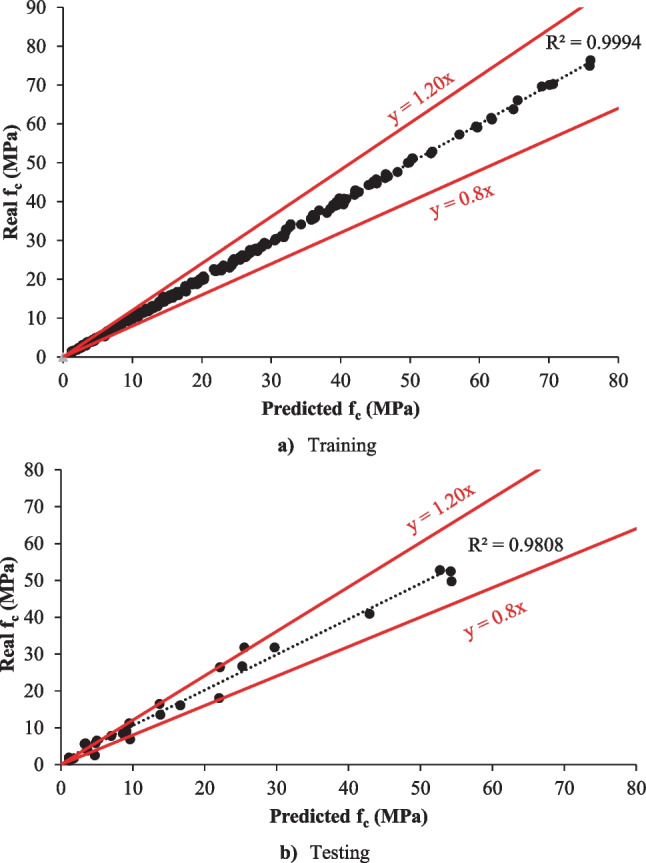
The training and testing results of the LSBoost model. **a** Training. **b** Testing

**Fig. 9:**
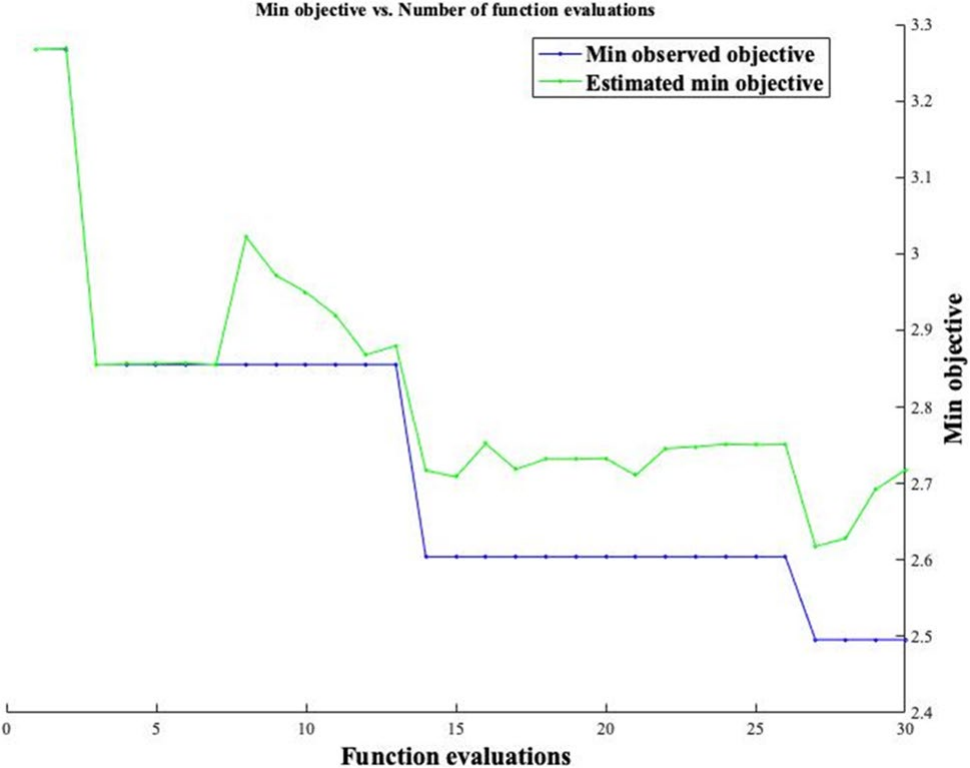
The minimum objective number of function evaluations of the LSBoost model

**Fig. 10:**
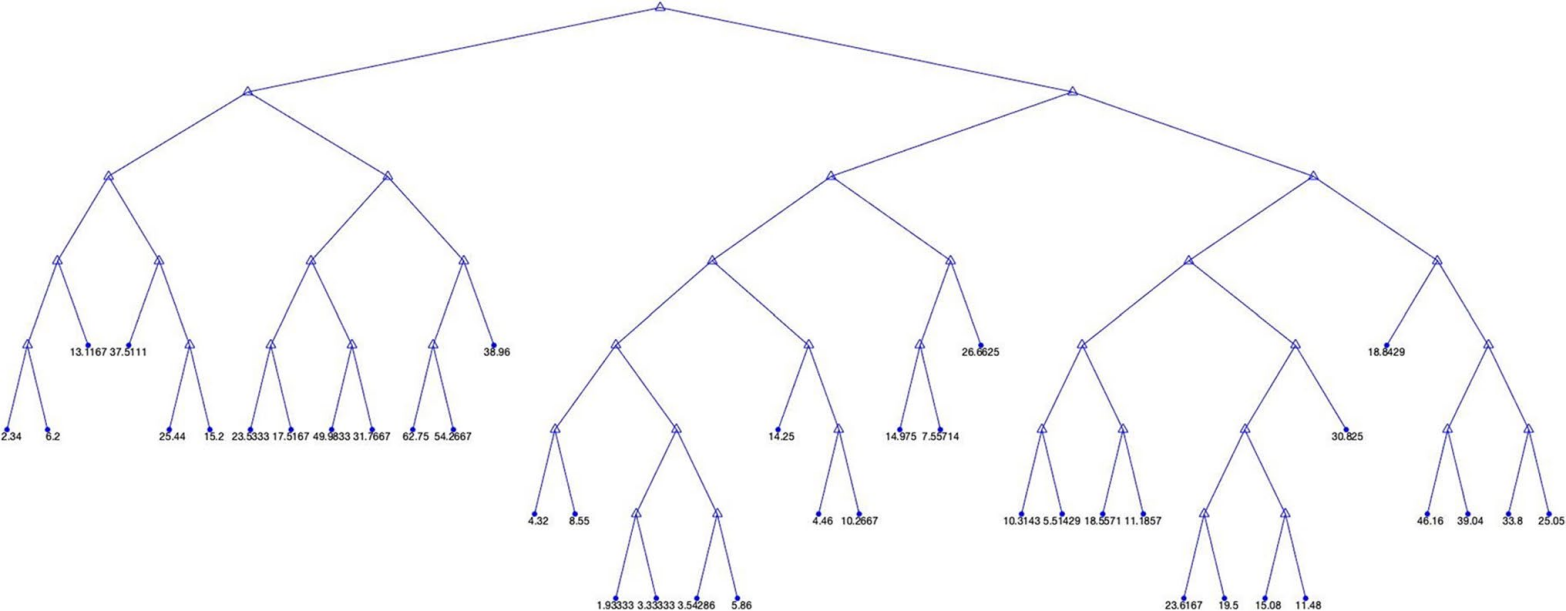
Tree view of LSBoost model

In this current study, the LSBoost model predicted the *f*_c_ of eco-friendly geopolymer concrete. The automatic hyperparameter optimization in the LSBoost model was used. The number of ensemble learning cycles, learning rate, and the maximum number of splits resulting from hyperparameter optimization were obtained as 498, 0.10478, and 24, respectively. The guess results of the LSBoost algorithm, the minimum objective number of function evaluations, and the tree view for the *f*_c_ are shown in Figs. 8, 9, and 10.

**Fig. 11:**
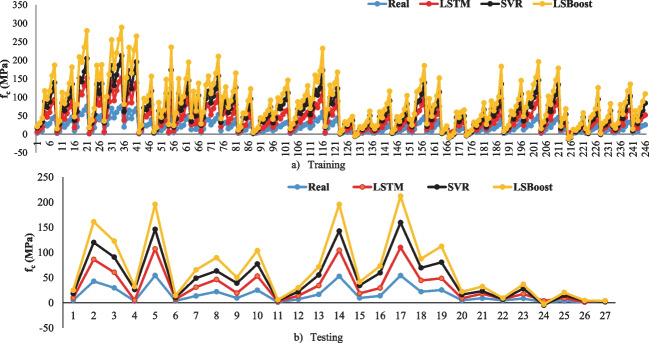
The comparison of all models for training and testing.** a** Training. **b** Testing

As can be seen in Fig. 8, the LSBoost model in the training and testing phase estimated the *f*_c_ with 99.94% and 98.08%, respectively. As in other algorithms, the NRMSE, MSE, PSNR, MAPE, and RMSE were used in assessing the LSBoost model. The MSE, PSNR, RMSE, NRMSE, and MAPE values in the training phase were obtained as 0.1594, 56.1057, 0.3993, 0.0053, and 2.3980, respectively. The MSE, PSNR, RMSE, NRMSE, and MAPE values for the testing phase were computed 5.5103, 40.7190, 2.3474, 0.0441, and 19.5143, respectively. The minimum objective number of function evaluations of the LSBoost model is displayed in Fig. 9. It can be seen from Fig. 9 that the min. observed objective was closer to the estimated min. objective. Also, the tree view of the LSBoost model is shown in Fig. 10. Although the LSBoost model guessed the *f*_c_ with higher accuracy than the SVR and MLR algorithms, it estimated the *f*_c_ with lower accuracy than the deep learning model. Zhang and Xu (2022) carried out a study to forecast the elastic modulus of concrete using the LSBoost model. They used the results of normal and high-strength concrete. They found that the recommended model forecasted the normal and high-strength concrete with 97.59% and 97.395 accuracy, respectively. Adamu et al. (2022) used the SVR, Gaussian Process Regression, MLR, and LSboost models to guess the flexural strength of concrete incorporating the calcium carbide and nano-silica. They expressed that the GPR model was superior to other models in the prediction of flexural strength. The comparison of all models is given in Fig. 11.

**Fig. 12:**
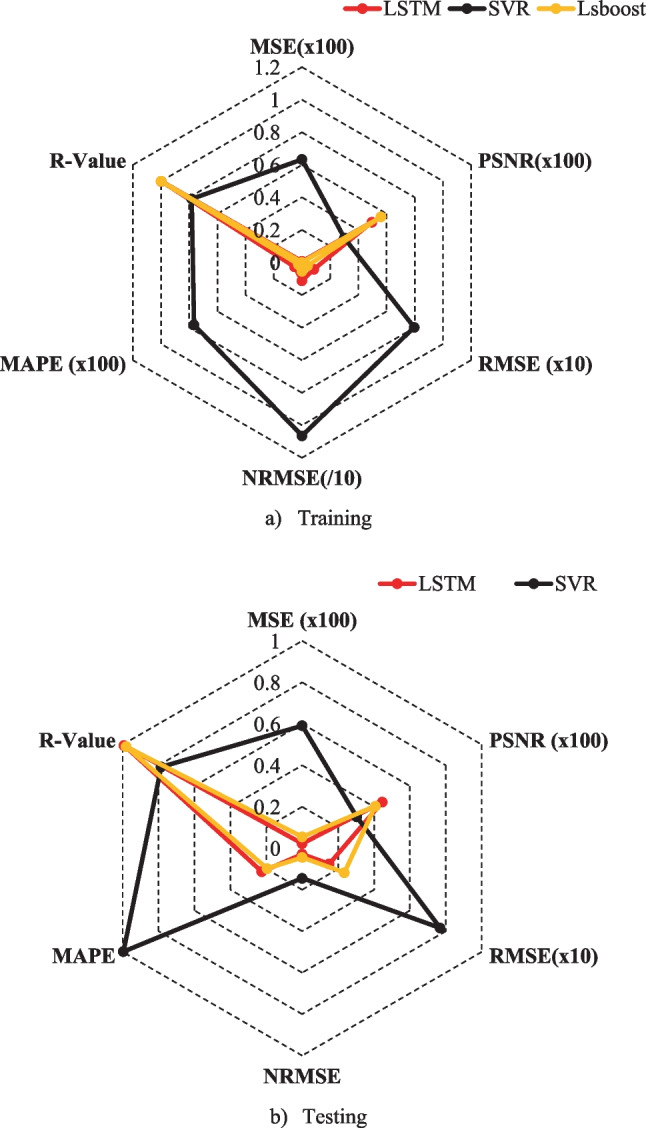
The radar chart comparisons of all metrics of the models.** a** Training. **b** Testing

**Fig. 13:**
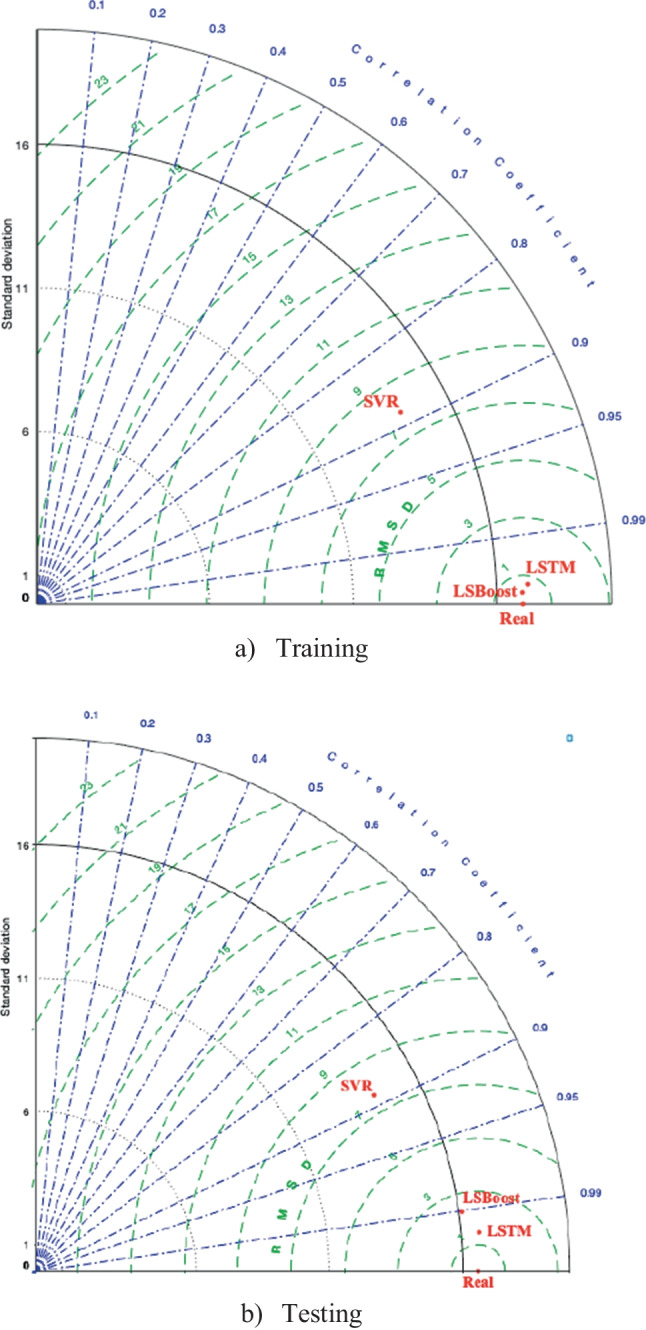
Taylor diagrams of the models. **a** Training. **b** Testing

It can be seen from Fig. 11 that the LSTM approach was closer to the real *f*_c_ values than other algorithms in both the testing and training stages. Thus, it can be said that the LSTM approach had a better predictive ability. The radar chart comparisons of prediction metrics of all models are given in Fig. 12. Figure 12a shows that the LSBoost model carried out a better performance than the LSTM and SVR algorithms because the lower MAPE, the higher *R*-value, the lower MSE, the lower RMSE values, the lower NRMSE, and the higher PSNR were obtained from the LSBoost model. Also, the metrics of the LSTM and LSBoost models are very close to each other. Figure 12b shows that the higher PSNR, the higher R-value, the lower MAPE, the lower NRMSE, the lower RMSE, and the lower MSE values were obtained from the LSTM model in the testing phase. So, the LSTM model showed better performance than the LSBoost and SVR models. Figure 13 illustrates the Taylor diagram for the training and testing phase. When the Taylor diagram is examined, the best predictor model in the testing phase was the LSTM model. Also, the best predictive model in the training stage was the LSboost model, but the LSboost model and LSTM model made very close estimations during the training phase.

### The results of sensitivity analysis

This study applied a sensitivity analysis to calculate the influence of the input variables. The sensitivity analysis (*r*_*ij*_) results for the *f*_c_ of eco-friendly geopolymer concrete are given in Fig. 13.

**Fig. 14:**
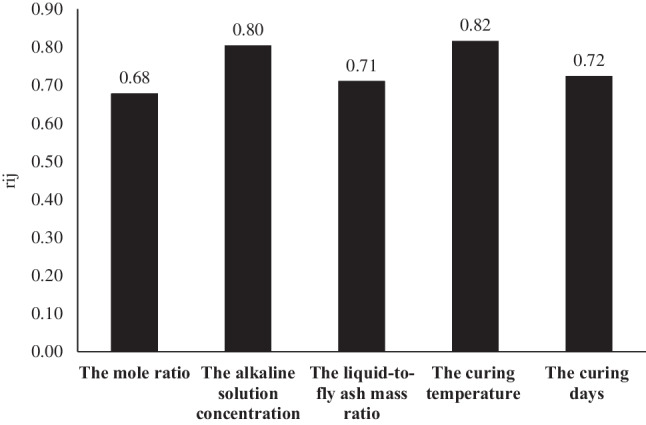
The sensitivity analysis results

It is shown in Fig. 14 that the curing temperature (0.82) was the most important variable on the *f*_c_ of eco-friendly geopolymer concrete. Tanyildizi and Yonar (2016) examined the effect of the curing temperature on the properties of fly ash-based geopolymer concrete with PVA fiber after being subjected to high temperatures. They kept the samples at 60, 80, and 100 °C. They said that the curing temperature was effective on compressive strength, and 60 °C was the best temperature. Abdullah et al. (2011) studied the differences in the alkaline solution concentration, the curing temperature, the liquid-to-fly ash mass ratio, and the mole ratio on the mechanical features of fly ash-based geopolymer concrete. They stated that the curing temperature had a major effect on the *f*_c_. Bai et al. (2023) studied the effect of curing temperature on eco-friendly geopolymer concrete. They stated that changing the curing temperature affects the *f*_c_ of the composite. They also stated that the *f*_c_ increased as it prevented crack formation in the geopolymer composite due to maintaining a certain moisture level during curing. Saludung et al. (2023) studied the effect of curing conditions on geopolymer composites. They showed that the curing condition has a substantial effect on the characteristics of the unexposed geopolymer composite. Gopalakrishna and Dinakar (2023) examined the *f*_c_ of geopolymer composites under different curing temperatures. They reported that as thermal curing conditions increase, the polymerization of the geopolymer composite accelerates, and thus the *f*_c_ increases. In this current study, results similar to the results in the literature were obtained.

## Conclusions

This study estimated the *f*_c_ of eco-friendly geopolymer concrete using the SVR, deep LSTM, LSBoost, and MLR models. Furthermore, the effect of the input variables in the algorithms was identified by the sensitivity analysis. The results of the *f*_c_ of eco-friendly geopolymer concrete were obtained from the literature. The results of 273 samples for *f*_c_ were used in the algorithms. The results obtained from this current study are given as follows:

- The deep LSTM model forecasted the *f*_c_ of eco-friendly geopolymer concrete with 99.23% accuracy, while the *f*_c_ of eco-friendly geopolymer concrete was forecasted using the SVR, LSBoost, and MLR models with 78.22%, 98.08%, and 88.03% accuracy, respectively. Furthermore, an ANN model in the literature estimated the same data with 96.1% accuracy, respectively. According to these results, the deep LSTM model forecasted the *f*_c_ of eco-friendly geopolymer concrete with higher accuracy than the LSBoost, SVR, MLR, and ANN models.
- The sensitivity analysis found that the most important input variable for the *f*_c_ of eco-friendly geopolymer concrete was the curing temperature.
- As a result of the results obtained in this research, guessing the compressive strength of geopolymer concrete with high accuracy using the deep LSTM model better serves the practical construction needs regarding the strength design of concrete. Also, it is thought that the application of deep learning-based approaches as a user-friendly interface to directly predict the mixture ratios required to obtain the desired compressive strength in structures will be useful for the construction industry. Thus, it will help develop sustainable and eco-friendly structures without time-consuming and costly experiments. Because of this superiority of the deep LSTM model, it can be recommended to be used in forecasting the properties of eco-friendly concrete or mortar.

## Data Availability

There are no repository data, and all data are present in the paper.
